# Is There Room for SERMs or SARMs as Alternative Therapies for Adult Male Hypogonadism?

**DOI:** 10.1155/2020/9649838

**Published:** 2020-01-21

**Authors:** Vito A. Giagulli, Andrea Silvestrini, Carmine Bruno, Vincenzo Triggiani, Alvaro Mordente, Antonio Mancini

**Affiliations:** ^1^Internal Medicine, Geriatrics, Endocrinology and Rare Disease, Interdisciplinary Department of Medicine, University of Bari, School of Medicine, Policlinico, Bari, Italy; ^2^Dipartimento di Scienze Biotecnologiche di Base, Cliniche Intensivologiche e Perioperatorie, Università Cattolica del Sacro Cuore, Fondazione Policlinico Universitario A Gemelli IRCCS, Rome, Italy; ^3^Dipartimento di Medicina e Chirurgia Traslazionale, Università Cattolica del Sacro Cuore, Fondazione Policlinico Universitario A Gemelli IRCCS, Rome, Italy

## Abstract

Hypogonadotropic hypogonadism (HH) can be sustained by organic or functional alterations of the hypothalamic-pituitary-testicular axis. Functional HH is related to systemic alterations, such as obesity or chronic inflammatory diseases, but could contribute to a negative course of the illness. For such situation, according to results obtained in infertile women, the administration of selective estrogen receptor modulators (SERMs) has been proposed in males too, with positive results on both metabolic and sexual function. This class of medications increases gonadotropin levels via antagonism to the estrogenic receptor; similar biological effects are also exerted by aromatase inhibitors (AIs), despite different mechanism of action. After a brief review of trials regarding SERMs and AIs use in male HH, we describe the structure and function of the androgen receptor (AR) as a basis for clinical research about compounds able to bind to AR, in order to obtain specific effects (SARMs). The tissue selectivity and different metabolic fate in comparison to testosterone can potentiate anabolic versus androgenic effects; therefore, they might be a valid alternative to testosterone replacement therapy avoiding the negative effects of testosterone (i.e., on prostate, liver, and hematopoiesis). Trials are still at an early phase of investigation and, at the moment, the application seems to be more useful for chronic disease with catabolic status while the validation as replacement for hypogonadism requires further studies.

## 1. Introduction

Hypogonadotropic hypogonadism, also known as secondary hypogonadism, is the most common form of hypogonadism in adult and elderly man [1], related to an absolute or relative defective secretion of gonadotropin-releasing hormone (GnRH) by the hypothalamus and/or gonadotropin secretion by the pituitary gland. It recognizes two main etiologies: the organic and the functional one. The organic form is characterized by a strong genetic stigma; expansive lesions of the hypothalamic-pituitary region, traumatic events, or, more commonly, infectious or infiltrative diseases can cause it. On the contrary, the functional form appears to be acquired and underpinned by multiple metabolic and inflammatory mechanisms [2]. Based on that evidence, most of the International Societies in the field stated that Testosterone replacement therapy (TRT) should be considered only for the organic forms, independently of the patients' age at the time of onset.

As functional hypogonadism is concerned, specific treatments for the underlying conditions inducing the T decrease (i.e., obesity, metabolic syndrome, diabetes mellitus, and so on) have been suggested [3, 4]. In these conditions, hypogonadism is related to a progressive worsening of the disease, despite an adequate therapy (for instance, the failure of diet in obese men) [5], contributing to the evolution by an unfavourable vicious circle. In addition, given the concerns about the benefit of TRT (for instance, in younger men willing to maintain their fertility) or the not fully proven safety of TRT for a long time, especially in fragile elderly subjects, other strategies have been object of investigation, for instance those that can activate the androgen receptor (AR) present in specific target tissues.

Hence, according to the results obtained in infertile women treated with either selective estrogen receptor modulators (SERMs) (i.e., clomiphene citrate) or aromatase inhibitors (AIs) (i.e., anastrozole or letrozole), which proved to enhance the serum levels of luteinizing hormone (LH) and follicle-stimulating hormone (FSH), men with secondary functional hypogonadism were treated with the same drugs in order to increase the endogenous T levels [6]. On the contrary, both SERMs and AIs maintain the fertility, avoiding the block of spermatogenesis, as TRT does [7, 8].

The knowledge about androgen receptors also allowed to develop another class of drugs, directly acting at this receptor, but with differential effects in comparison to T supplementation (selective androgen receptor modulators, SARMs). In fact, they are considered as a new promising class of compounds to have anabolic effects for a number of clinical indications, without causing negative effects on the prostate, red blood cells, and cardiovascular system. In addition, they could be employed in several functional limitations, often associated with chronic disease, frailty, cancer cachexia, and osteoporosis.

The aim of the present review is to update information about both SERMs and SARMs in the treatment of male hypogonadism. Many clinical trials are reported on SERMs, while the field of SARMs is still largely at an early phase of investigation since pharmacological aspects of a number of new molecules have been recently made available. After a brief review of trials concerning SERMs use, we focus the attention on the androgen receptor as the basis of the expanding field of SARMs. It is known that TRT can have undesired effects as discussed in the following; therefore, the search for such compounds could be promising as an alternative therapy.

## 2. SERMs and AIs: Mechanism of Action and Clinical Practice Uses

### 2.1. Mechanism of Action

SERMs and AIs are able to elicit the secretion of LH and FSH with different mechanism: a direct action at the hypothalamus (SERMs), where they block estrogen receptors (such as clomiphene citrate, CC), or an indirect one (AIs), decreasing plasma oestrogens levels via a block of aromatase [7]. LH acts, consequently, at the testes to increase T secretion, while FSH may positively coregulate Sertoli cells function and the spermatogenesis. Due to their activity, SERMs and AIs are also considered as indirect androgen doping due to their ability to increase T, being thus prohibited both in amateur and professional athletes [9].

The raise in LH and FSH, however, requires a normal functional hypothalamic-pituitary-testis axis that can be elicited independently of the age of the subjects treated. Indeed, a retrospective study indicated that a testicular volume <14 cc and LH levels >6 UI/ml might be considered as the strong predictors of a good response to CC treatment [10], highlighting that all forms of primary hypogonadism and the secondary organic ones might be unresponsive to that therapy [11]. In young adults and elderly men, CC (50 mg/day), anastrozole (1 mg/day), and letrozole (2.5 mg/die) were able to raise plasma levels of LH and FSH, highlighting the key inhibiting role of oestrogens levels on gonadotropins secretion at the hypothalamus-pituitary level [12].

Furthermore, since the effects of both drugs were similar in young and elderly men, it can be hypothesized that an increased negative feedback by endogenous oestrogens is not instrumental in age-related decline of Leydig cell function in elderly men [13]. Similarly, letrozole (2.5 mg/day for 6 months) was able to promote the development of puberty in boys with constitutional delay of growth and puberty, further emphasizing the functional role of the impairment of the hypothalamic-pituitary-testis axis in this clinical condition [6].

### 2.2. Impact on Circulating Steroid Levels

Although both SERMs and AIs can raise gonadotropin levels, they induce a different pattern of circulating steroid levels, as suggested by an important difference in the serum T/17*β* oestradiol (E2) ratio, depending on the kind of drug employed [14]. Depending on their action, in fact, AIs can reduce circulating oestrogens, while CC does not. On the other hand, it is commonly known that E2 has a crucial role in male reproduction and sexual function [15] as well as in the brain functions and the metabolic activity. Indeed, in a hormonal milieu characterized by a normal/high T/E2 ratio by means of letrozole (2.5 mg/day) therapy, positive behavioural effects can be observed (fear and risk taking) in healthy adult men [16].

### 2.3. Metabolic Effects

A normal/high T/E2 ratio has proven to be able to reduce insulin resistance by lowering insulin and glucose levels, without body composition changing in nonobese adult men [17], highlighting a direct and specific role of T in coregulating the insulin secretion and activity [18]. CC also shares positive metabolic effects. In a recent double-blind placebo-controlled study, CC (25 mg/day) improved fasting glycaemia, insulin levels, and homeostasis model assessment estimate of insulin-resistance (HOMA-IR) in obese men with functional secondary hypogonadism, regardless of their glucose tolerance state (impaired glucose tolerance or type 2 diabetes mellitus) [19]. Interestingly, similar results were obtained by chronic T supplementation in a mixed population of normal men or male subjects affected by secondary hypogonadism. This study, in fact, showed that TRT enhances the body composition by increasing lean mass at the expense of fat mass, indicating the crucial role of T as a coregulator of body composition [20].

### 2.4. SERMs and AIs as Therapy for Hypogonadism

In addition to hormonal and metabolic outcomes, there is a large amount of evidence about the symptomatic improvement of hypogonadal men on CC therapy. As a matter of fact, even in comparison with TRT, the treatment with CC was able to improve the A questionnaire and satisfaction in hypogonadal men [21]. Conversely, few studies with inconclusive data have been carried out on CC and AIs effect on erectile dysfunction (ED) in men so far [22]. Guay and coll, in fact, evaluated CC effect in secondary hypogonadic men and ED, showing that CC improved sexual function. At variance with these results, both a retrospective study evaluating 77 hypogonadal and infertile men treated with CC [23] and a double-blind placebo-controlled study involving 17 subjects did not show any improvement in sexual function during CC therapy [24]. Similarly, Leder and coll. did not find any significant difference in ED assessed by the International Index of Erectile Function during anastrozole (1 mg/day) therapy in a placebo-controlled study conducted in elderly men [25].

The common side effects reported with the use of both classes of drugs are scarce since this topic in particular is less explored when compared with TRT. They include hot flashes, insomnia, and weight gain for AIs and gynecomastia, dizziness, and headache for CC. However, AIs could determine a reduction in bone mineral density owing to their suppressive effect on serum oestrogens [26], whereas CC therapy caused a markedly lower incidence of secondary polycythaemia in comparison with TRT in hypogonadal men [27].

Anyway, both classes of drugs have proven to be capable to reactivate the hypothalamus-pituitary-testis axis independently of age (pubertal and adult men) in all clinical conditions without an organic damage but with only a partial and/or functional block of gonadotropin secretion. Moreover, given that they do not have relevant side effects, AIs and SERMs seem to be an attractive and quite safe alternative therapy for male secondary hypogonadism and for infertile men without organic damages at the hypothalamic-pituitary-testis axis. However, it is desirable that more studies involving large cohorts of patients and a longer period of observation should be carried out, with the aim to draw definitive evidence about those therapies, especially in the light to compare SERMs and AIs results with those obtained with T substitution in men with secondary hypogonadism.

## 3. SARMs: Biochemical Action and Therapeutic Effects

Active androgens, T and its 5*α*-hydroxy-end metabolite [dihydrotestosterone (DHT)], carry out their main effects by means of AR. Selective androgen receptor modulators (SARMs) are compounds which can bind AR similarly to active androgens, still retaining their androgen effect and displaying a tissue-selective activation of AR without showing those important negative effects of T supplementation especially in the prostate tissue, red blood cells, high-density lipoprotein (HDL), and cardiovascular system. Thus, they might be especially used in adult and elderly men suffering from the impairment of important organs such as heart, kidney, prostate, bone, and muscle. To improve the knowledge on SARMs mechanism of action, the characteristics of AR are briefly updated.

### 3.1. Androgen Receptor: Structure and Function

The androgen receptor (AR), also known as NR3C4 (nuclear receptor subfamily 3, group C, gene 4), is a member of the steroid receptor subfamily of the nuclear receptors (NRs) superfamily. It plays pivotal roles in the development and maintenance of homeostasis of the reproductive, musculoskeletal, cardiovascular, immune, neural, and haematopoietic tissues.

In human beings, the androgen receptor (MW 110 kDa; 919-920 amino acids) is encoded by the AR gene located on the long arm chromosome X at the locus Xq11- Xq12 [28, 29]. Like other members of the NRs superfamily, AR comprises three main functional domains: (1) an amino-terminal domain (NTD, residues 1–555) that shows little sequence homology with other steroid receptors and contains two independent activation regions (i.e., activation function 1 and activation function 5) essential for the transcriptional activation of AR; (2) a DNA-binding domain (DBD, residues 555–623) which is highly conserved among the steroid receptors and consists of two zinc finger motifs that recognise specific DNA consensus sequences, thus playing a crucial role in AR binding to androgen response elements (AREs); and (3) a carboxyl-terminal domain (CTD, residues 665–919) which includes the ligand-binding domain (LBD) and contains the ligand‐binding pocket (LBP).

The DBD and LBD regions are connected by a flexible hinge region (residues 628–669) [30], called nuclear localization signal (NLS), that is, a lysine rich sequence and is important for nuclear localization of the AR receptor and is also a major target site for posttranslational modifications such as acetylation, methylation, ubiquitylation, and phosphorylation [31].

AR is expressed in several reproductive (breast, testes, prostate, ovary, and endometrium) and nonreproductive tissues (hair follicles, bone, brain, liver, and cardiovascular) [32].

Several different mutations of the AR gene have been identified so far. Some of them might lower its function leading to the androgen insensitivity syndrome [33]; they encompass pathologic conditions which occur with different clinical phenotypes: spinal and bulbar muscular atrophy and partial or complete androgen resistance (i.e., Morris syndrome). Conversely, some other mutations of AR can be activating as it does in prostate cancer.

Immunohistochemistry studies have revealed that AR has been expressed in 75% of breast tumours. Consequently, AR is suggested as a new marker and potential therapeutic target in the treatment of patients against breast cancer [34].

**Figure 1:**
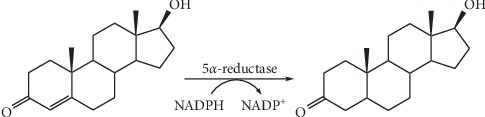
Testosterone (left) is converted by 5*α*-reductase into 5*α*-dihydrotestosterone (DHT) (right) in a NADPH-dependent redox reaction.

When an active androgen (e.g., testosterone; T) crosses the cell membrane, it either directly binds the inactive AR or is converted into the more potent dihydrotestosterone (DHT) by 5-*α*-reductase (Figure 1). Moreover, DHT binds the inactive form of AR strongly than T. In fact, intracellular DHT is a more potent androgenic agonist than T, and its existence in specific reproductive tissues (external genitalia, seminal vesicle, and prostate) is required for organ development and their correct functions. Additionally, at low circulating androgen levels, DHT binding to AR is favourite in respect to T [35].

**Figure 2:**
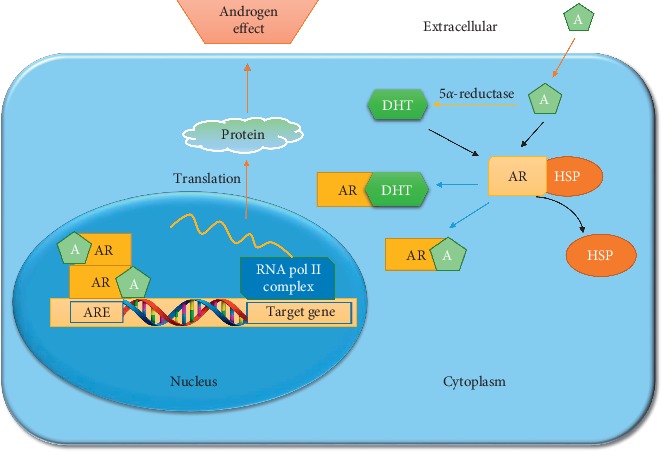
Illustration of the AR signalling pathway. When A enters into the cell, it is converted into the more potent DHT by 5*α*-reductase and binds to the ligand-binding pocket of AR forming AR-A and AR-DHT complexes, respectively. Consequently, the AR-A or AR-DHT is translocated into the nucleus with help of other proteins to form the AR-dimer (or AR-DHT dimer, not shown) that is able to bind the ARE region in DNA to regulate the expression of downstream genes thus initiating the transcription of specific protein that exert the androgen effect.

The biochemical mechanisms of androgenic activity include several sequential steps that are illustrated in Figure 2. Briefly, in the absence of androgen, AR is localized in the cytoplasm as an inactive complex with heat shock proteins (e.g., HSP70 and HSP90) and chaperone proteins, which help to maintain the apo state of the AR competent to bind the ligand. When androgen (A) enters the cell, it directly binds the inactive AR. Due to the binding of androgens to AR, it undergoes a conformational change which leads to the dissociation of HSP and other proteins from the inactive AR that is converted in the active form [36]. The activated AR (i.e., active AR which binds with A or DHT) complex is translocated to the nucleus and dimerizes as a AR homodimer (AR-dimer) that acts as a transcriptional regulator activating the gene expression by binding to the specific sequence, the androgen response element (ARE), site in DNA [37]. Homodimerization of AR is mediated by the dimerization box located in the DBD region to achieve an intermolecular DBD-DBD link and N/C-terminal interactions through the FXXLF motif [38]. The AR-DNA-binding domain is responsible for the binding to DNA that takes place through a palindromic consensus sequence 5′-GGTACAnnnTGTTCT-3′, the so-called androgen response element (ARE) that is recognized by the AR-dimer.

Moreover, the AR-dimer can form signalling complexes with coregulated proteins to enhance or depress transcription of the target gene (i.e., modulates gene transcription of the AR-target genes), thus exerting androgen effect. AR-activated genes comprise KLK3 that encode the prostate‐specific antigen (PSA), KLK2, FKBP5, transmembrane protease serine 2 (TMPRSS2), and several other transcripts [39].

While androgens are important for normal development of various tissues, under definite circumstances they also could prompt pathological effects on the prostate, the liver, and the myocardium. Interestingly, the AR agonists were proven to be beneficial for anabolic deficiencies. The ideal AR ligand for the treatment of anabolic deficiencies should be tissue selective. Such AR ligands were defined as selective androgen receptor modulators (SARMs). SARMs were first described and subsequently developed to facilitate selective tissue activation of the AR [40]. Several of the SARMs designed so far possess a nonsteroidal structure and have the ability to activate the AR in the muscle and bone, with a reduced activation of the AR in the prostate. Thus, SARMs may serve as therapeutic options for treatment of numerous diseases, including muscle weakness, osteoporosis, and breast cancer.

### 3.2. SARMs: Biochemistry

Based on this knowledge about the androgen receptor, a large field of research has been developed about compounds able to bind AR in order to obtain specific effects (SARMs). Some of these are obtained by modification of the steroid structure, but others are nonsteroidal; it is an old concept, the myotrophic/androgenic index [41] as a major tool to evaluate androgen potency. The aim of this kind of investigations is to find compounds with anabolic activity without negative effects of androgenic activity. Moreover, nonsteroidal SARMs are not metabolized by aromatase or 5-*α*-reductase.

The ideal profile of an effective SARM, for the treatment of hypogonadism, should include a comfortable administration (oral and once daily administration), ability to stimulate physical and psychological sexual items, anabolic effects (body composition, muscle strength, and bone growth), avoiding undesirable side effects, such as liver toxicity, fluid retention, and blood pressure increase, overstimulation of erythropoiesis, and induction of gynecomastia [42]. Moreover, the effects on the prostate gland should be carefully monitored, avoiding adverse effects associated to proliferative stimulation which are related to 5-*α*-reductase; therefore, the different metabolism in comparison to T is among the desired characteristics. The aim to further ameliorate anabolic effects should be on the other hand preferentially pursued for the extended indications to SARM therapy, especially osteoporosis in elderly men [43, 44], glucocorticoid-induced osteoporosis [43], HIV sarcopenia and cancer cachexia [45], anaemia, and muscular dystrophies [42, 46]. These aspects could be also extended to women.

It is known that the idea to develop SARM, modifying the T structure, is not new (steroidal SARMs) [47]. The main strategies were

- The 17-*α*-alkyl substitution, with extension of its half-life and the possibility of oral administration; the hepatotoxicity and unfavourable effects on lipid pattern limited this approach;
- Removal of the 19-methyl group, with enhancement of anabolic activity (nandrolone series); also metabolism is influenced since these derived molecules are not aromatizable but are transformed by 5-*α*-reductase in less potent androgens; a further 7-*α*-alkyl substitution increases anabolic effects and reduces effects on prostate;
- Esterification of the 17-*β*-hydroxyl group, with extension of duration of action;
- A 17-*α*-alkyl substitution has been realized also starting by DHT; substitution of a second carbon with oxygen increases its stability and anabolic activity, without aromatization to oestrogens.

The modern era started with the synthesis on nonsteroidal SARMs [40, 42, 48–51]. The first generation of ligands was obtained by structural modifications of arylpropionamide analogs, bicalutamide, and hydroxyflutamide.

Then, different classes of SARMs have been obtained, including propionamide, quinolinone, tetrahydroquinoline, bicyclic-hydantoin, pyrazoline, imidazolpyrazole, benzimidazole, and aniline [52]. As discussed before, AR has a widespread distribution in reproductive and nonreproductive tissues and has a complex regulation related to recruitment and activation of coactivators and corepressors, followed by tissue-specific gene activation. Interestingly, three hypotheses are discussed about selectivity of SARMs [47]:

- A coactivator hypothesis, based on differences in transcriptional activity, related to the role played by activators and repressors;
- A conformational hypothesis, based on the induction of changes in ligand-binding domains, modulation of surface topology, and subsequent protein-protein interaction (both at cytosolic level with nongenomic effects and at regulators of gene expression);
- Differences in tissue distribution and metabolic fate of the different SARMs.

### 3.3. Biological Effects and Clinical Applications

The activity of SARMs is confirmed by both in vitro and in vivo models. A well-known model is the demonstration of AR-dependent agonist activity in castrated rats (Hershberger assay).

From a pathophysiological point of view, the interest is focused on catabolic conditions, such as muscle atrophy as observed in sepsis, ageing, cancer, AIDS [53], and bone catabolism in osteoporosis; the effects of nonsteroidal SARM have been explored in castrated male Sprague Dawley rats and compared with glucocorticoid-induced muscle atrophy [53]. SARMs, but also T, counteracted the upregulation of ubiquitin ligases. Molecular mechanism in the other model (castration) was instead a blunting effect on other signalling, such as upregulation of ligases but especially downregulation of IGF-1. Recently, we described the interaction IGF-1 gonadal function [54]. Interestingly, another promising field of investigation involves the activation of AR signalling in the skeletal muscle, which is also indirect, mediated by upregulation of follistatin and a consequent cross-communication from the Wnt-*β* catenin pathway to the TGF*β*-SMAD pathway. These interactions are specific for the muscle, and therefore could be a strategy to a further development of anabolic drugs [47, 55].

In addition, bone metabolism is greatly influenced by gonadal milieu. It is widely accepted that patients with low levels of estradiol present bone metabolism disorders and increased risk of fractures [56, 57]. This phenomenon could depend on a reduced aromatization of testosterone into estradiol. Different studies support this view showing severe male osteoporosis in patients affected by mutations of the estrogen receptor or mutations of the aromatase [58, 59]. However, both testosterone and estradiol seem to be fundamental for bone metabolism, even with different mechanisms. Various evidences suggested a dominant role of estrogen on bone resorption, whereas both estrogen and testosterone are involved in maintaining bone formation [60].

In a gonadectomised model of skeletally-mature male rats, an increase in biochemical markers of bone reabsorption rapidly develops; anyhow, SARMs administration counteracted these effects, as confirmed by biochemical studies but also histomorphometric and mechanical analyses [61]. These studies could be the basis for a clinical application in the treatment of osteoporosis.

### 3.4. Clinical Trials

A number of clinical trials are ongoing as shown in Table 1.

Among the published trials, a 12-week double-blind, placebo-controlled, phase II clinical trial evaluated GTx-024 (enobosarm) effects in 120 healthy elderly men (age > 60 years) and postmenopausal women; a significant dose-dependent increase in total lean body mass evaluated by DEXA was reported. Also, physical function and insulin resistance improved. Adverse effect did not differ in comparison to the placebo. Interestingly, the abovementioned effects persisted although there was a reduction of serum total testosterone due to the significant lowering plasma levels of SHBG [62].

Another study evaluated safety, tolerability, pharmacodynamics, and pharmacokinetics of Gsk2881078 in healthy young men. A decrease in HDL and SHBG was described with dose-related effects. Adverse effects were mild [63].

The same drug was tested in a randomized placebo-controlled parallel group repeat dose, a dose escalation study in healthy older men; this study confirms good tolerability without serious adverse event. Transient elevations of alanine amino transferase were observed; the main focus was the increase of lean mass assessed by DEXA and MRI cross-sectional thigh scans [64].

In the above-reported studies, however, a greater response was observed in women.

The purpose of most cited studies is the treatment of unfavourable metabolic status in patients with systemic diseases; use of SARMs as replacement therapy for hypogonadism is not well defined since in this case also androgenic properties should be maintained; the objective is still to identify molecules with good androgenic and myotropic properties only avoiding tissue-selective undesired actions; also the modality of administration should be comfortable and well accepted. Indeed, in a recent placebo-controlled study that lasted 21 days, conducted in 76 healthy young men (21–50 years) who were randomized to placebo or oral different doses of LGD-4033 (0.1, 0.3, or 1.0 mg per day) showed a dose-dependent increase of lean body mass and presented a significant reduction of serum HDL, indicating a potential negative effect on the lipoprotein profile [65]. With the aim to reduce the above negative effect on HDL levels that can allow to also use SARMs in elderly hypogonadal men, a recent transdermal SARM (LY305) has been proposed in the preclinical study [66]. LY305, in fact, was found to be a safe and well-tolerated transdermal nonsteroidal androgen modulator which offers a potential therapeutic option for elderly patients suffering from signs and symptoms of hypogonadism.

## 4. Conclusion

Although there are different mechanisms of action, both SERMs and SARMs can find an appropriate role in treating hypogonadism condition in adult and elderly men. While SERMs can reactivate the hypothalamic-pituitary-testis axis whenever it is functionally blocked owing to chronic diseases (metabolic and/or not metabolic conditions), SARMs, overcoming the hypothalamic-pituitary-testis axis function, act by directly stimulating AR. On the contrary, both families of drugs appear not to cause important side effects as TRT could do during chronic therapy. However, concerning SERMs, there are several observational and randomized studies which have confirmed their use in clinical setting, while about SARMs there are some preclinical studies which point out interesting results in adult hypogonadal men suffering from severe form of catabolic processes such as muscle atrophy, cachexia, and severe osteoporosis. Based on this evidence, although preclinical data suggest a positive outcome for SARMs, further clinical trials with larger sample sizes and randomized design are needed before using them in the clinical practice particularly in elderly hypogonadal men.

## Figures and Tables

**Table 1:** Selective androgen receptor agonists (SARMs): ongoing clinical trials.

Status	Study title	Conditions	Interventions	Locations
Not yet recruiting	Multimodality Intervention for Function and Metabolism in SCI	(i) Spinal cord injuries	(i) Drug: SARM (ii) Behavioral: hybrid exercise	

Active, not recruiting	Study to Evaluate the Safety and Efficacy of 13 weeks of the Selective Androgen Receptor Modulator (SARM) GSK2881078 in Chronic Obstructive Pulmonary Disease (COPD)	(i) Cachexia	(i) Drug: GSK2881078 (ii) Placebo	USA

Active, not recruiting	Pembrolizumab and Enobosarm in Treating Patients With Androgen Receptor Positive Metastatic Triple Negative Breast Cancer	(i) Androgen receptor positive (ii) Estrogen receptor negative (iii) HER2/neu negative (iv) And 3 more	(i) Drug: enobosarm (ii) Pembrolizumab	USA

Recruiting	A Selective Androgen Receptor Modulator for Symptom Management in Prostate Cancer	(i) Prostate cancer	(i) Drug: LY2452473 (ii) Drug: placebo	USA

Data from https://clinicalltrials.gov.

## References

[1] Tajar A., Forti G., O’Neill T. W. (2010). Characteristics of secondary, primary, and compensated hypogonadism in aging men: evidence from the European male ageing study. *The Journal of Clinical Endocrinology & Metabolism*.

[2] Grossmann M., Matsumoto A. M. (2017). A perspective on middle-aged and older men with functional hypogonadism: focus on holistic management. *The Journal of Clinical Endocrinology & Metabolism*.

[3] Bhasin S., Brito J. P., Cunningham G. R. (2018). Testosterone therapy in men with hypogonadism: an endocrine society∗ clinical practice guideline. *The Journal of Clinical Endocrinology & Metabolism*.

[4] Yeap B. B., Grossmann M., McLachlan R. I. (2016). Endocrine society of Australia position statement on male hypogonadism (part 2): treatment and therapeutic considerations. *Medical Journal of Australia*.

[5] Apovian C. M., Aronne L. J., Bessesen D. H. (2015). Pharmacological management of obesity: an endocrine society clinical practice guideline. *The Journal of Clinical Endocrinology & Metabolism*.

[6] Varimo T., Huopio H., Kariola L. (2019). Letrozole versus testosterone for promotion of endogenous puberty in boys with constitutional delay of growth and puberty: a randomised controlled phase 3 trial. *The Lancet Child & Adolescent Health*.

[7] Lo E. M., Rodriguez K. M., Pastuszak A. W., Khera M. (2018). Alternatives to testosterone therapy: a review. *Sexual Medicine Reviews*.

[8] Chua M. E., Escusa K. G., Luna S., Tapia L. C., Dofitas B., Morales M. (2013). Revisiting oestrogen antagonists (clomiphene or tamoxifen) as medical empiric therapy for idiopathic male infertility: a meta-analysis. *Andrology*.

[9] Handelsman D. J. (2008). Indirect androgen doping by oestrogen blockade in sports. *British Journal of Pharmacology*.

[10] Mazzola C. R., Katz D. J., Loghmanieh N., Nelson C. J., Mulhall J. P. (2014). Predicting biochemical response to clomiphene citrate in men with hypogonadism. *The Journal of Sexual Medicine*.

[11] Liel Y. (2017). Clomiphene citrate IN the treatment of idiopathic or functional hypogonadotropic hypogonadism IN men: a case series and review of the literature. *Endocrine Practice*.

[12] Tenover J. S., Bremner W. J. (2019). The effects of normal aging on the response of the pituitary-gonadal axis to chronic clomiphene administration in men. *Journal of Andrology*.

[13] T’Sjoen G. G., Giagulli V. A., Delva H., Crabbe P., De Bacquer D., Kaufman J.-M. (2005). Comparative assessment in young and elderly men of the gonadotropin response to aromatase inhibition. *The Journal of Clinical Endocrinology & Metabolism*.

[14] Helo S., Ellen J., Mechlin C. (2015). A randomized prospective double-blind comparison trial of clomiphene citrate and anastrozole in raising testosterone in hypogonadal infertile men. *The Journal of Sexual Medicine*.

[15] Schulster M., Bernie A., Ramasamy R. (2016). The role of estradiol in male reproductive function. *Asian Journal of Andrology*.

[16] Goudriaan A. E., Lapauw B., Ruige J. (2010). The influence of high-normal testosterone levels on risk-taking in healthy males in a 1-week letrozole administration study. *Psychoneuroendocrinology*.

[17] Gibb F. W., Homer N. Z. M., Faqehi A. M. M. (2016). Aromatase inhibition reduces insulin sensitivity in healthy men. *The Journal of Clinical Endocrinology & Metabolism*.

[18] Lapauw B., Ouwens M., ‘t Hart L. M. (2010). Sex steroids affect triglyceride handling, glucose-dependent insulinotropic polypeptide, and insulin sensitivity: a 1-week randomized clinical trial in healthy young men. *Diabetes Care*.

[19] Pelusi C., Giagulli V. A., Baccini M. (2017). Clomiphene citrate effect in obese men with low serum testosterone treated with metformin due to dysmetabolic disorders: a randomized, double-blind, placebo-controlled study. *PLoS One*.

[20] Corona G., Giagulli V. A., Maseroli E. (2016). Therapy of endocrine disease: testosterone supplementation and body composition: results from a meta-analysis study. *European Journal of Endocrinology*.

[21] Ramasamy R., Scovell J. M., Kovac J. R., Lipshultz L. I. (2014). Testosterone supplementation versus clomiphene citrate for hypogonadism: an age matched comparison of satisfaction and efficacy. *Journal of Urology*.

[22] Guay A. T., Jacobson J., Perez J. B., Hodge M. B., Velasquez E. (2003). Clomiphene increases free testosterone levels in men with both secondary hypogonadism and erectile dysfunction: who does and does not benefit?. *International Journal of Impotence Research*.

[23] Chandrapal J. C., Nielson S., Patel D. P. (2016). Characterising the safety of clomiphene citrate in male patients through prostate-specific antigen, haematocrit, and testosterone levels. *BJU International*.

[24] Guay A. T., Bansal S., Heatley G. J. (1995). Effect of raising endogenous testosterone levels in impotent men with secondary hypogonadism: double blind placebo-controlled trial with clomiphene citrate. *The Journal of Clinical Endocrinology & Metabolism*.

[25] Leder B. Z., Rohrer J. L., Rubin S. D., Gallo J., Longcope C. (2004). Effects of aromatase inhibition in elderly men with low or borderline-low serum testosterone levels. *The Journal of Clinical Endocrinology & Metabolism*.

[26] Burnett-Bowie S.-A. M., McKay E. A., Lee H., Leder B. Z. (2009). Effects of aromatase inhibition on bone mineral density and bone turnover in older men with low testosterone levels. *The Journal of Clinical Endocrinology & Metabolism*.

[27] Wheeler K. M., Smith R. P., Kumar R. A., Setia S., Costabile R. A., Kavoussi P. K. (2017). A comparison of secondary polycythemia in hypogonadal men treated with clomiphene citrate versus testosterone replacement: a multi-institutional study. *Journal of Urology*.

[28] Lubahn D. B., Joseph D. R., Sar M. (1988). The human androgen receptor: complementary deoxyribonucleic acid cloning, sequence analysis and gene expression in prostate. *Molecular Endocrinology*.

[29] Gelmann E. P. (2002). Molecular biology of the androgen receptor. *Journal of Clinical Oncology*.

[30] Wang Q., Lu J., Yong E. L. (2001). Ligand- and coactivator-mediated transactivation function (AF2) of the androgen receptor ligand-binding domain is inhibited by the cognate hinge region. *Journal of Biological Chemistry*.

[31] Clinckemalie L., Vanderschueren D., Boonen S., Claessens F. (2012). The hinge region in androgen receptor control. *Molecular and Cellular Endocrinology*.

[32] Kimura N., Mizokami A., Oonuma T., Sasano H., Nagura H. (1993). Immunocytochemical localization of androgen receptor with polyclonal antibody in paraffin-embedded human tissues. *Journal of Histochemistry & Cytochemistry*.

[33] Batista R. L., Costa E. M. F., de Santi Rodrigues A. (2018). Androgen insensitivity syndrome: a review. *Archives of Endocrinology and Metabolism*.

[34] Venema C. M., Bense R. D., Steenbruggen T. G. (2019). Consideration of breast cancer subtype in targeting the androgen receptor. *Pharmacology & Therapeutics*.

[35] Zhou Z. X., Lane M. V., Kemppainen J. A., French F. S., Wilson E. M. (1995). Specificity of ligand-dependent androgen receptor stabilization: receptor domain interactions influence ligand dissociation and receptor stability. *Molecular Endocrinology*.

[36] Cano L. Q., Lavery D. N., Bevan C. L. (2013). Mini-review: foldosome regulation of androgen receptor action in prostate cancer. *Molecular and Cellular Endocrinology*.

[37] Prescott J., Coetzee G. A. (2006). Molecular chaperones throughout the life cycle of the androgen receptor. *Cancer Letters*.

[38] Xu D., Zhan Y., Qi Y. (2015). Androgen receptor splice variants dimerize to transactivate target genes. *Cancer Research*.

[39] Chen C. D., Welsbie D. S., Tran C. (2004). Molecular determinants of resistance to antiandrogen therapy. *Nature Medicine*.

[40] Dalton J. T., Mukherjee A., Zhu Z., Kirkovsky L., Miller D. D. (1998). Discovery of nonsteroidal androgens. *Biochemical and Biophysical Research Communications*.

[41] Kicman A. T. (2008). Pharmacology of anabolic steroids. *British Journal of Pharmacology*.

[42] Negro-Vilar A. (1999). Selective androgen receptor modulators (SARMs): a novel approach to androgen therapy for the new millennium. *The Journal of Clinical Endocrinology & Metabolism*.

[43] Orwoll E. S., Klein R. F. (1995). Osteoporosis in men. *Endocrine Reviews*.

[44] Snyder P. J., Peachey H., Hannoush P. (1999). Effect of testosterone treatment on body composition and muscle strength in men over 65 years of age. *The Journal of Clinical Endocrinology & Metabolism*.

[45] Bhasin S., Storer T. W., Asbel-Sethi N. (1998). Effects of testosterone replacement with a nongenital, transdermal system, androderm, in human immunodeficiency virus-infected men with low testosterone levels ^1^. *The Journal of Clinical Endocrinology & Metabolism*.

[46] Bagatell C. J., Bremner W. J. (1996). Androgens in men—uses and abuses. *New England Journal of Medicine*.

[47] Bhasin S., Jasuja R. (2009). Selective androgen receptor modulators as function promoting therapies. *Current Opinion in Clinical Nutrition and Metabolic Care*.

[48] Edwards J. P., West S. J., Pooley C. L. F., Marschke K. B., Farmer L. J., Jones T. K. (1998). New nonsteroidal androgen receptor modulators based on 4-(trifluoromethyl)-2(1H)-pyrrolidino[3,2-g] quinolinone. *Bioorganic & Medicinal Chemistry Letters*.

[49] Hamann L. G., Mani N. S., Davis R. L., Wang X.-N., Marschke K. B., Jones T. K. (1999). Discovery of a potent, orally active, nonsteroidal androgen receptor agonist: 4-Ethyl-1,2,3,4-tetrahydro-6- (trifluoromethyl)-8-pyridono[5,6- g]- quinoline (LG121071). *Journal of Medicinal Chemistry*.

[50] Miner J. N., Chang W., Chapman M. S. (2007). An orally active selective androgen receptor modulator is efficacious on bone, muscle, and sex function with reduced impact on prostate. *Endocrinology*.

[51] He Y., Yin D., Perera M. (2002). Novel nonsteroidal ligands with high binding affinity and potent functional activity for the androgen receptor. *European Journal of Medicinal Chemistry*.

[52] Zhang X., Lanter J. C., Sui Z. (2009). Recent advances in the development of selective androgen receptor modulators. *Expert Opinion on Therapeutic Patents*.

[53] Jones A., Hwang D.-J., Narayanan R., Miller D. D., Dalton J. T. (2010). Effects of a novel selective androgen receptor modulator on dexamethasone-induced and hypogonadism-induced muscle atrophy. *Endocrinology*.

[54] Mancini A., Bruno C., Palladino A., Vergani E., Giacchi E., Singh R. (2019). GH–IGF1 axis in spermatogenesis and male fertility. *Molecular Signaling in Spermatogenesis and Male Infertility*.

[55] Singh R., Bhasin S., Braga M. (2009). Regulation of myogenic differentiation by androgens: cross talk between androgen receptor/*β*-catenin and follistatin/transforming growth factor-*β* signaling pathways. *Endocrinology*.

[56] Szulc P., Munoz F., Claustrat B. (2001). Bioavailable estradiol may be an important determinant of osteoporosis in men: the minos study 1. *The Journal of Clinical Endocrinology & Metabolism*.

[57] Barrett-Connor E., Mueller J. E., von Mühlen D. G., Laughlin G. A., Schneider D. L., Sartoris D. J. (2000). Low levels of estradiol are associated with vertebral fractures in older men, but not women: the rancho bernardo study 1. *The Journal of Clinical Endocrinology & Metabolism*.

[58] Belgorosky A., Guercio G., Pepe C., Saraco N., Rivarola M. A. (2009). Genetic and clinical spectrum of aromatase deficiency in infancy, childhood and adolescence. *Hormone Research in Paediatrics*.

[59] Morishima A., Grumbach M. M., Simpson E. R., Fisher C., Qin K. (1995). Aromatase deficiency in male and female siblings caused by a novel mutation and the physiological role of estrogens. *The Journal of Clinical Endocrinology & Metabolism*.

[60] Khosla S. (2013). Pathogenesis of age-related bone loss in humans. *The Journals of Gerontology Series A: Biological Sciences and Medical Sciences*.

[61] Rosen J., Negro-Vilar A. (2002). Novel, non-steroidal, selective androgen receptor modulators (SARMs) with anabolic activity in bone and muscle and improved safety profile. *Journal of Musculoskeletal and Neuronal Interactions*.

[62] Dalton J. T., Barnette K. G., Bohl C. E. (2011). The selective androgen receptor modulator GTx-024 (enobosarm) improves lean body mass and physical function in healthy elderly men and postmenopausal women: results of a double-blind, placebo-controlled phase II trial. *Journal of Cachexia, Sarcopenia and Muscle*.

[63] Clark R. V., Walker A. C., Andrews S., Turnbull P., Wald J. A., Magee M. H. (2017). Safety, pharmacokinetics and pharmacological effects of the selective androgen receptor modulator, GSK2881078, in healthy men and postmenopausal women. *British Journal of Clinical Pharmacology*.

[64] Neil D., Clark R. V., Magee M. (2018). GSK2881078, a SARM, produces dose-dependent increases in lean mass in healthy older men and women. *The Journal of Clinical Endocrinology & Metabolism*.

[65] Basaria S., Collins L., Dillon E. L. (2013). The safety, pharmacokinetics, and effects of LGD-4033, a novel nonsteroidal oral, selective androgen receptor modulator, in healthy young men. *The Journals of Gerontology: Series A*.

[66] Krishnan V., Patel N. J., Mackrell J. G. (2018). Development of a selective androgen receptor modulator for transdermal use in hypogonadal patients. *Andrology*.

